# Identification and QTL Analysis of Flavonoids and Carotenoids in Tetraploid Roses Based on an Ultra-High-Density Genetic Map

**DOI:** 10.3389/fpls.2021.682305

**Published:** 2021-06-11

**Authors:** Bixuan Cheng, Huihua Wan, Yu Han, Chao Yu, Le Luo, Huitang Pan, Qixiang Zhang

**Affiliations:** ^1^Beijing Key Laboratory of Ornamental Plants Germplasm Innovation & Molecular Breeding, National Engineering Research Center for Floriculture, Beijing Laboratory of Urban and Rural Ecological Environment, Engineering Research Center of Landscape Environment of Ministry of Education, Key Laboratory of Genetics and Breeding in Forest Trees and Ornamental Plants of Ministry of Education, School of Landscape Architecture, Beijing Forestry University, Beijing, China; ^2^Beijing Advanced Innovation Center for Tree Breeding by Molecular Design, Beijing Forestry University, Beijing, China

**Keywords:** *Rosa*, flower color, whole genome sequencing, genetic linkage map, QTL analysis

## Abstract

Roses are highly valuable within the flower industry. The metabolites of anthocyanins, flavonols, and carotenoids in rose petals are not only responsible for the various visible petal colors but also important bioactive compounds that are important for human health. In this study, we performed a QTL analysis on pigment contents to locate major loci that determine the flower color traits. An F_1_ population of tetraploid roses segregating for flower color was used to construct an ultra-high-density genetic linkage map using whole-genome resequencing technology to detect genome-wide SNPs. Previously developed SSR and SNP markers were also utilized to increase the marker density. Thus, a total of 9,259 markers were mapped onto seven linkage groups (LGs). The final length of the integrated map was 1285.11 cM, with an average distance of 0.14 cM between adjacent markers. The contents of anthocyanins, flavonols and carotenoids of the population were assayed to enable QTL analysis. Across the 33 components, 46 QTLs were detected, explaining 11.85–47.72% of the phenotypic variation. The mapped QTLs were physically clustered and primarily distributed on four linkage groups, namely LG2, LG4, LG6, and LG7. These results improve the basis for flower color marker-assisted breeding of tetraploid roses and guide the development of rose products.

## Introduction

Roses are one of the most important ornamental plants worldwide, with a high value in various areas such as cut flowers, gardens and the food and medical industries. The genus *Rosa* contains approximately 200 species, but only 7–10 of them have contributed to the breeding of modern roses (Scariot et al., 2006). After a long history of hybridization, most modern roses have valuable characters that have formed from a complex genetic background at the tetraploid level. Currently, the number of modern rose cultivars has reached approximately 40,000 in number. However, the inheritance patterns of many rose traits remain unclear owing to their high heterozygosity, various ploidy levels and extreme intrageneric variation (Liorzou et al., 2016).

Flower color is one of the most significant traits of ornamental plants, and it also plays an important role in improving the aesthetic and economic values of roses. The appearance of flower color is largely affected by the various pigments synthesized by plants. The types of these pigments primarily include flavonoids, carotenoids and betalains, with flavonoids and carotenoids contributing the most to formation of flower color. Flavonols and anthocyanins are particularly important classes of flavonoids. Flavonols are highly prevalent in flower petals of various colors and play key roles in attracting insect pollinators. Flavonols primarily turn plants to yellow, while anthocyanins can provide orange, red, pink and blue colors to plants (Tanaka et al., 2008). Carotenoids primarily determine the yellow and orange colors of flowers and are also essential to human nutrition and health (Mayne, 1996).

Because of the complicated genetic background in roses, understanding the process of pigment synthesis in roses is particularly challenging. However, molecular markers and genetic mapping can provide an effective way to analyze the mechanism by which multiple genes affect variation in pigment regulation at the population level. Substantial progress has been made in the genetic mapping of tetraploid roses.

The first tetraploid rose genetic map was constructed by Rajapakse et al. (2001) using amplified fragment length polymorphism (AFLP) and simple-sequence repeat (SSR) markers. Over the last 20 years, major tetraploid populations include the double pseudo test cross population GGFC, which was used to study flower morphological traits based on AFLP and SSR markers (Guterman et al., 2002; Gar et al., 2011). The K5 population, developed from tetraploid cut roses, has been used to study the inheritance of multiple traits, including prickle density (Koning-Boucoiran et al., 2012), petal number (Koning-Boucoiran et al., 2012; Bourke et al., 2018a), resistance to powdery mildew (Koning-Boucoiran et al., 2009, 2012) and anthocyanin metabolism (Gitonga et al., 2016). The YS population, which was derived from the ancient rose ‘Yunzheng Xiawei’ and the modern rose ‘Sun City’ (Yu et al., 2015), has been shown to segregate for petal number, flower size and flower color. Lastly, a tetraploid climbing rose population (Zurn et al., 2018) has been used to study resistance to black spot.

High-throughput sequencing technologies are powerful tools that can greatly increase the density and quality of genetic linkage maps. Whole-genome sequencing (WGS) technology can be used to compare each individual genome to a reference genome and thus provide considerable nucleotide sequence information in a short time. Thus, WGS tools enable researchers to analyze the genetic mechanism of traits and finely map important genes at the genomic level (Smulders et al., 2019). These approaches have the advantages of being fast, efficient and high yielding, and after years of development, the associated costs have been dramatically reduced. In recent years, several rose high-density genetic maps based on genotyping sequencing and single nucleotide polymorphism (SNP) array technology have been constructed (Vukosavljev et al., 2016; Bourke et al., 2017; Yan et al., 2018). However, compared with these techniques, WGS can develop much more data than SNP arrays and thus, obtain more comprehensive genomic information. As a powerful tool, WGS is widely used in scientific research. Its enormous sequencing datasets provide a solid basis for analyzing individual differences, population evolution and genetic mechanisms.

Over the last 20 years, the number of marker types and overall marker density have increased substantially in rose genetic maps, with the total genome coverage also increasing (Vijay et al., 2021). However, the breeding and production of modern roses are primarily conducted with tetraploid cultivars. Differences in the ploidy level remain a major issue in rose QTL mapping, and it has resulted in low efficiency of applications based on diploid maps. Thus, with the rapid development of sequencing technology and analysis methods, QTL mapping at the tetraploid level poses significant advantages.

In this study, an ultra-high-density tetraploid rose genetic map was constructed using whole genome resequencing technology. Based on this map, we performed QTL analysis with pigment content data of flavonoids and carotenoids. Our study combined genomics and metabolomics to finely map flower-color-related loci to a genetic linkage map of tetraploid roses and analyze the genetic factors that influence pigment content in rose petals.

## Materials and Methods

### Mapping Population

The YS population used in this study was previously described by Yu et al. (2015). The ancient Chinese garden rose cultivar ‘Yunzheng Xiawei’ (YX) was used as the female parent, and the modern rose cultivar ‘Sun City’ (SC) was used as the male parent. Both parents are tetraploid. A total of 187 individuals were selected from among the F_1_ progeny. Both parents and the resulting progeny were planted at the Xiao Tangshan Experimental Base of the National Engineering Research Center for Floriculture, Beijing, China. The parents and the YS population were grown under the same environment with regular irrigation and fertilization.

### Pigment Content Extraction and Analysis

The petals of parents and offspring were collected at full bloom, immediately frozen in liquid nitrogen and stored at −80°C. The flavonoids and carotenoids were extracted by HPLC and LC-MS as described by Wan et al. (2018).

#### Extraction and Analysis of Flavonoids

A total of 0.15–0.25 g samples were weighed, ground and then extracted in 0.9 mL of solvent (methanol/water/formic acid/trifluoroacetic acid, 70:27:2:1, *v*/*v*/*v*/*v*) in an ultrasonic cleaner (KQ-250DA, Jiangsu, China). The HPLC analyses were conducted using a Waters 2695 HPLC system connected with a 996-photodiode array detector (Waters, Milford, MA, United States). The supernatant was injected into an XBridge BEH C18 reversed-phase column (150 mm × 4.6 mm, 2.5 μm, Waters). The mobile phase was composed of 0.5% aqueous formic acid (A) and acetonitrile (B). The gradients were programmed as follows: 0 min, 5% B; 5 min, 10% B; 30 min, 19% B; 50 min, 40% B; 50.01-60 min, 5% B. The column temperature was 25°C, the detection wavelengths of anthocyanin and flavonol were 520 and 350 nm, respectively. All samples were extracted in triplicate. LC-MS analysis was performed on the same HPLC system as described above using the parameters described by Wan et al. (2018).

Two-milligram flavonoid standards (cyanidin 3,5-diglucoside, pelargonidin 3,5-diglucoside, quercetin 3-*O*-glucoside, kaempferol 3-*O*-rutinoside, kaempferol 3-*O*-glucoside, and kaempferol) were diluted to six concentration gradients. The peak areas corresponding to each concentration of the flavonoid standards were recorded, and standard curves were drawn to obtain linear regression equations. Then, the peak area of the identified flavonoid pigment was substituted into the regression equation, and the content of the corresponding substance in the solution was calculated.

#### Extraction and Analysis of Carotenoids

After the samples were quickly ground, 0.2 g of each sample was weighed and promptly added to 4 mL of methanol, which was then shaken for 20 min. Afterward, 2 mL of NaCl solution (10%, w/v) was added, followed by 30 s of manual shaking. The organic phase in the upper layer was collected after the samples were allowed to stand. The mixed organic phase was then dried and saponified. A volume of 1 mL of NaCl solution (10%, w/v) was added to the saponified sample, followed by 30 s of manual shaking. Samples were allowed to stand for 5 min and then incubated with 1 mL of *n*-hexane/ether (3:1, *v*/*v*) for 1 h, after which the supernatant was finally evaporated to dryness and stored at −80°C. All of the extraction processes were conducted in darkness or yellow light, and the temperature did not exceed 23°C. The dried carotenoids were dissolved in 1 mL of organic solvent (methanol/methyl tert-butyl ether = 1:1, *v*/*v*), which was then filtered and used for HPLC analysis. Three mobile phases were used: A, methanol; B, methyl tert-butyl ether; C, ultra-pure water. The gradients were set as follows: 0–2 min, 95% A + 5% C, 10 min, 95% A + 3% B + 2% C; 21 min, 95% A + 5% B; 27 min, 90% A + 10% B; 37 min, 70% A + 30% B; 40 min, 50% A + 50% B; 40.01–50 min, 95% A + 5% C. The injection volume was 20 μL; the column temperature was 25°C, and the detection wavelength of carotenoid was 450 nm. The mass spectrometry information was obtained by HPLC-microTOF-Q. All the samples were extracted in triplicate. An LC-MS analysis was performed on the same HPLC platform as noted above, and the parameters were the same as those described by Wan et al. (2018).

Carotenoid standards ([all-*E*]-β-carotene, [all-*E*]-violaxanthin, [all-*E*]-antheraxanthin, [all-*E*)]-zeaxanthin and [all-*E*]-lutein) were weighed and diluted with 10 mL of organic solvent (methanol: methyl tert-butyl ether = 1:1) to prepare standard solutions with six concentration gradients. The peak area corresponding to each concentration of the five types of carotenoid standards was recorded, and standard curves for the five types of standards were drawn. Corresponding linear regression equations were then obtained. The peak area of the identified carotenoids was substituted into the regression equation to calculate the content of each substance in the test solution.

### DNA Extraction

DNA was extracted from young leaves of the parents and the YS population using a Plant Genomic DNA Extraction Kit from TIANGEN (Beijing, China) according to the manufacturer’s instructions. Total DNA was extracted from each of three biological replicates. The concentration of DNA was measured using an ultraviolet spectrophotometer. After extraction, all DNA samples were stored at −20°C.

### SNP Detection and Genotyping

Whole-genome resequencing was used to detect genome-wide variants. After detection of the samples, the genomic DNA was segmented, and the qualified gDNA was selected for library construction. Following fragmentation, a 2100 Bioanalyzer platform (Agilent Technologies, Santa Clara, CA, United States) was used for quality control, and the library was prepared after end repair and 3′ A-tailing. After PCR amplification, the insert size was detected again using the Agilent 2100 Bioanalyzer. Finally, the high-quality samples were sequenced on an Illumina NovaSeq PE150 platform (Illumina, San Diego, CA, United States) using standard Illumina protocols.

The raw data were filtered as follows: (1) Cutadapt v2.3 (Martin, 2011) was used to cut the reads with N base calls at both ends or low-quality bases (quality score <20); (2) the reads with more than 5% N base calls were removed; (3) reads with more than 50% bases with a quality score <20 were removed. High-quality clean data were obtained after the quality check and filtering. Clean reads were then compared to the reference genome sequence of *Rosa chinensis* ‘Old Blush’ (Hibrand Saint-Oyant et al., 2018) using BWA software (Li and Durbin, 2010). SNPs were detected using Sentieon Haplotyper (Weber et al., 2016) based on the alignment results. The SNP markers were then genotyped under autotetraploid mode. Each locus was converted into a dosage value. The dosage values corresponding to genotypes of AAAA, AAAB, AABB, ABBB, and BBBB were 0, 1, 2, 3, and 4, respectively, with the genotypes of both parents and the offspring converted according to this rule.

### Linkage Map Construction

In addition to the SNP markers obtained by whole genome resequencing, we also added simple sequence repeats (SSRs) (Yu et al., 2015), and the SNPs that were previously developed by specific locus amplified fragment sequencing (SLAF-seq) (Yu et al., 2021) to the current map. Linkage map construction was performed using the polyploid mapping R package polymapR (Bourke et al., 2018b).

The dosage-scored data were imported into polymapR. The markers were filtered according the following exclusion criteria: (1) non-segregating markers; (2) markers with more than 5% missing data; and (3) samples with more than 1% missing data.

The parental linkage maps were first constructed, and then the consensus map was integrated afterward. The ‘cluster_SN_markers’ function was first used to cluster the (1,0) markers. According to the clustering result, we chose to add (2,0) markers and use the ‘bridgeHomologues’ function to obtain four clusters that corresponded to the four homologs of each chromosome. The remaining types of markers were assigned to each homolog and were ordered using the MDSMap package (Preedy and Hackett, 2016). After the parental maps were constructed, all the homolog information from two parents was combined, and the markers were ordered again to construct the integrated map. Finally, the integrated genetic map was used to generate phased linkage maps to conduct QTL analysis.

### QTL Analysis

The QTL analysis was conducted using composite interval mapping (CIM) with the R package polyqtlR (Bourke et al., 2018c). Phased maps and dosage marker information were imported into polyqtlR, together with the pigment content data. Identity-by-descent (IBD) probabilities for the population were estimated using polyOrigin (Zheng et al., 2020). Based on the IBD results, an initial QTL scan was performed with each set of phenotypic data. Significance thresholds of LOD values were determined using a genome-wide permutation test with 1,000 permutations (α = 0.05). The initial QTL results were outputted with peak positions and LOD values. The already-identified QTL positions were then included as genetic co-factors to reduce the amount of unwanted background variance. Further rounds of QTL scans were performed to determine other QTL positions and to check if multiple peaks within a single linkage group were indeed independent. The QTL results were confirmed and outputted when no new peaks could be identified. The visualization of QTL results was performed by polyqtlR in the R statistical computing environment (R Development Core Team, 2016). Markers located at the LOD peaks or closest to both ends of the QTL intervals were considered to be closely linked to the QTLs. The naming of QTLs was based on previously published guidelines (McCouch et al., 1997).

## Results

### Pigment Content Variation

#### Anthocyanin Content Variation

Four anthocyanin compounds were detected in the petals of F_1_ population: cyanidin 3,5-diglucoside (0.00–3403.36 μg/g fresh weight [FW]), pelargonidin 3,5-diglucoside (0.00–813.19 μg/g FW), pelargonidin 3-*O*-glucoside (0.00–312.69 μg/g FW), and cyanidin 3-*O*-glucoside (0.00–190.55 μg/g FW). The contents of anthocyanin in the F_1_ population ranged from 0.00 to 3,509.90 μg/g FW, and the coefficient of variation ranged from 148.56 to 367.58%. This indicated that the variation in anthocyanin metabolism in the F_1_ population was extremely high.

**FIGURE 1:**
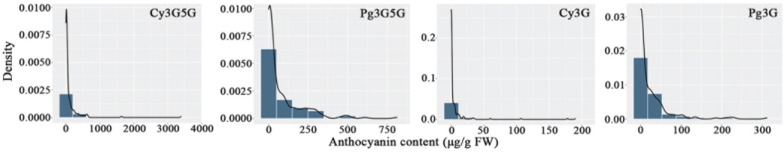
Frequency distribution of anthocyanin contents in the tetraploid rose YS population. Cy3G5G, cyanidin 3,5-diglucoside; Pg3G5G, pelargonidin 3,5-diglucoside; Cy3G, cyanidin 3-*O*-glucoside; Pg3G, pelargonidin 3-*O*-glucoside.

Eight characters related to anthocyanin content were recorded to perform further analyses (Table 1). There were extreme values of pigment content segregation in the F_1_ population, and the skewness of each of the eight characters was greater than 1, indicating a positive skew distribution. Further analysis of the frequency distribution showed that all the traits exhibited continuous variation in the mapping population (Figure 1), indicating these traits were quantitative traits controlled by polygenes and therefore suitable for QTL analysis.

**TABLE 1:** Statistical analysis of anthocyanins in the petals of parents and F_1_ progeny.

Traits	Compound	YX ♀	SC ♂	Min (μg/g FW)	Max (μg/g FW)	Average (μg/g FW)	*SD*	*S*	*K*	CV (%)
a1	Cyanidin 3,5-diglucoside	38.78	0	0	3403.36	113.08	300.56	8.01	80.65	265.8
a2	Pelargonidin 3,5-diglucoside	0	0	0	813.19	82.08	130.13	2.39	7.12	158.54
a3	Cyanidin 3-*O*-glucoside	0	0	0	190.55	5.83	21.44	6.98	53.6	367.58
a4	Pelargonidin 3-*O*-glucoside	0	0	0	312.69	23.88	44.43	3.46	15.01	186.05
a6	T3G5G	38.78	0	0	3403.36	195.16	370.94	4.87	33.78	190.07
a7	T3G	0	0	0	490.7	29.71	60.26	4.24	23.45	202.8
a8	TCy	38.78	0	0	3509.9	118.91	314.99	7.82	76.62	264.9
a9	TPg	0	0	0	945.36	105.96	157.42	2.16	5.7	148.56
a10	TA	38.78	0	0	3509.9	220.91	403.21	4.49	28.45	182.52

*YX, ‘Yunzheng Xiawei’ cultivar; SC, ‘Sun City’ cultivar; FW, fresh weight; SD, standard deviation; S, skewness; K, kurtosis; CV, coefficient of variation; T3G5G, total of cyanidin 3,5-diglucoside and pelargonidin 3,5-diglucoside; T3G, total of cyanidin 3-O-glucoside and pelargonidin 3-O-glucoside; TCy, total of cyanidin 3,5-diglucoside and cyanidin 3-O-glucoside; TPg, total of pelargonidin 3,5-diglucoside and pelargonidin 3-O-glucoside; TA, total anthocyanins.*

#### Flavonol Content Variation

**FIGURE 2:**
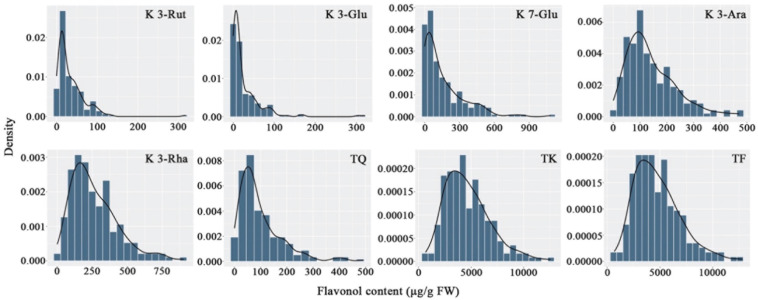
Frequency distribution of flavonol contents in the tetraploid rose YS population. K 3-Rut, kaempferol 3-*O*-rutinoside; K 3-Glu, kaempferol 3-*O*-glucoside; K 7-Glu, kaempferol 7-*O*-glucoside; K 3-Ara, kaempferol 3-*O*-arabinoside; K 3-Rha, kaempferol 3-*O*-rhamnoside; TQ, total quercetin; TK, total kaempferol; TF, total flavonol.

The flavonols in the petals of parents and F1 progeny were determined; the results are shown in (Table 2), and the frequency distribution is shown in (Figure 2). The contents of 20 flavonols and total quercetins, total kaempferols and total flavonols showed transgressive segregation. The coefficient of variation of 23 traits related to flavonol contents ranged from 44.85% to 191.52% and showed continuous variation in the F1 population, indicating that the traits were suitable for QTL analysis.

**TABLE 2:** Statistical analysis of flavonols in the petals of parents and progenies.

Traits	Identification	YX ♀	SC ♂	Min (μg/g FW)	Max (μg/g FW)	Average (μg/g FW)	*SD*	*S*	*K*	CV(%)
f1	Kaempferol 3-*O*-rhamnoside-7-*O*-glucoside	78.18	54.53	9.13	329.01	116.65	57.29	1.36	2.41	49.11
f2	Quercetin 3-*O*-glycoside	30.34	36.61	0	112.98	30.55	22.32	1.26	1.5	73.06
f3	Quercetin 7-*O*-glucoside	46.35	68.15	0	206.82	39.6	38.49	1.41	2.5	97.18
f4	Flavan-3-ol derivative	17.72	52.98	0	318.18	34.36	35.44	3.38	21.43	103.13
f5	Kaempferol 3-*O*-rutinoside	546.41	616.09	4.88	1610.01	342.45	242.2	1.65	4.25	70.73
f6	Kaempferol 3-*O*-glucoside	1158.53	581.24	26.66	2487.82	617.53	445.13	1.47	2.2	72.08
f7	Kaempferol 3-*O*-glucuronide	37.2	221.65	0	1083.25	159.89	184.22	1.82	4.21	115.22
f8	Kaempferol 3-*O*-(galloyl)-glucoside	140.24	148.09	0	471.24	141.73	92.62	1.18	1.37	65.35
f9	Quercetin 7-*O*-rhamnoside	0	61.37	0	359.84	28.08	53.78	3.31	13.26	191.52
f10	Kaempferol 3-*O*-xyloside	264.52	283.41	0	903.08	264.64	166.62	1.13	1.31	62.96
f11	Kaempferol 7-*O*-glucoside	0	1346.56	0	4285.06	911.83	932.66	1.12	0.83	102.28
f12	Kaempferol 3-*O*-arabinoside	51.48	321.46	0	1467.89	131.65	145.98	5.09	39.82	110.88
f13	Kaempferol 3-*O*-hexoside	73.48	288.68	0	693.25	173.78	121.47	1.12	1.53	69.9
f14	Kaempferol 3-*O*-rhamnoside	1296.6	752.01	26.53	4062.17	1520.16	681.78	0.7	0.45	44.85
f15	Kaempferol 3-*O*-glycoside 1	10.51	56.18	0	316.31	38.07	42.04	2.57	11.1	110.41
f16	Kaempferol 3-*O*-glycoside 2	0	73.5	0	670.52	59.49	78.54	3.33	19.82	132.03
f17	Kaempferol 7-*O*-(galloyl)-glucoside	0	33.61	0	217.79	38.98	47.25	1.54	2.29	121.22
f18	Kaempferol 3-*O*-glycoside 3	0	114.06	0	214.41	46.05	47.75	1.61	2.33	103.69
f19	Kaempferol 3-*O*-(*p*-coumaroyl)-glucoside	20.88	0.98	0	74.95	13.72	16.07	1.33	1.47	117.1
f20	Kaempferol	48.45	71.32	0	702.79	82.75	88.5	3.37	16.22	106.94
f30	TQ	76.69	166.13	0	490.78	98.23	85.08	1.86	4.24	86.61
f31	TK	3726.47	4963.37	667.34	12544.08	4659.38	2159.75	0.9	0.87	46.35
f32	TF	3803.16	5129.5	686.21	12813.8	4757.61	2200.42	0.91	0.91	46.25

*YX, ‘Yunzheng Xiawei’ cultivar; SC, ‘Sun City’ cultivar; FW, fresh weight; SD, standard deviation; S, skewness; K, kurtosis; CV, coefficient of variation; TQ, total quercetins; TK, total of kaempferols; TF, total flavonols.*

#### Carotenoid Content Variation

**FIGURE 3:**
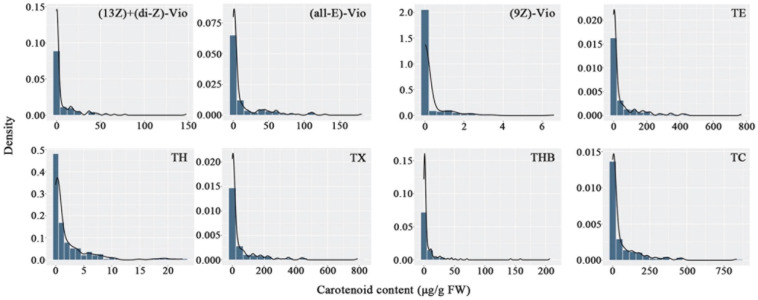
Frequency distribution of carotenoid contents in the tetraploid rose YS population. (13*Z*) + (di-*Z*)-Vio; (13*Z*) + (di-*Z*)-violaxanthin; (all-*E*)-Vio, (all-*E*)-violaxanthin; (9*Z*)-Vio, (9*Z*)-violaxanthin; TE, total epoxycarotenoids; TH, total hydroxycarotenoids; TX, total xanthophylls; THB, total hydrocarbons; TC, total carotenoids.

Carotenoids in the petals of parents and F_1_ progeny were also determined (Table 3). There were 16 carotenoids detected in the petals of the male parent SC, and no carotenoids were found in the female parent YX. A total of 16 carotenoid compounds were identified in the F1 progeny. The carotenoid contents in the petals of different progenies ranged from 0.00 to 467.12 μg/g FW, and the coefficient of variation ranged from 166.86 to 821.44%. In total, 21 traits were recorded for further analysis. The frequency distribution showed that all 21 traits exhibited continuous variation in the mapping population (Figure 3), indicating that these traits were quantitative traits controlled by polygenes and therefore suitable for QTL analysis.

**TABLE 3:** Statistical analysis of carotenoids in the petals of parents and F_1_ progeny.

Traits	Identification	YX ♀	SC ♂	Min (μg/g FW)	Max (μg/g FW)	Average (μg/g FW)	*SD*	*S*	*K*	CV (%)
c1	(13Z)-violaxanthin	0	15.09	0	5.88	0.29	0.87	3.69	14.75	298.65
c2	(13Z) + (di-Z)-violaxanthin	0	147.81	0	77.77	6.99	13.55	2.63	7.45	193.83
c3	(all-E)-violaxanthin	0	181.02	0	129.29	14.06	25.58	2.4	5.62	181.98
c4	(13/13′Z)-antheraxanthin	0	3.63	0	11.6	0.11	0.91	11.5	142.48	821.44
c5	(All-E)-luteoxanthin	0	17.01	0	13.12	1.67	3.04	2.29	4.57	181.54
c6	(13/13′Z)-neoxanthin	0	1.97	0	2.02	0.16	0.34	3.48	13	212.09
c7	(9Z)-violaxanthin	0	311.46	0	176.98	18.56	36.62	2.64	6.61	197.27
c8	(All-E)-antheraxanthin	0	40.38	0	66.23	3.47	8.7	4.4	23.28	250.34
c9	Xanthophyll 1	0	1.93	0	10.5	0.31	1.08	6.19	48.4	343.67
c11	(All-E)-zeaxanthin	0	16.42	0	22.54	1.89	3.67	3.41	13.36	193.79
c12	(9/9′Z)-lutein epoxide	0	27.04	0	16.87	1.32	2.65	2.96	10.29	200.2
c13	(All-E)-cryptoxanthin 5,6-epoxide	0	3	0	2.25	0.13	0.33	3.58	15.02	251.55
c14	(All-E)-alloxanthin	0	1.54	0	2.19	0.13	0.37	3.55	13.56	285.67
c15	(13Z)-β-carotene	0	6.26	0	6.03	0.74	1.28	2.42	5.39	173.17
c16	(All-E)-β-carotene	0	33.99	0	199.42	7.63	22.22	6.3	44.96	290.98
c17	(9Z)-β-carotene	0	5.12	0	5.14	0.5	0.87	2.71	8.6	173.4
c20	TE	0	771.03	0	437.05	47.42	87.99	2.51	6.21	185.57
c21	TH	0	19.3	0	22.62	2.28	3.93	2.96	10.09	171.84
c22	TX	0	790.33	0	451.96	49.7	91.33	2.51	6.25	183.76
c23	THB	0	45.37	0	207.01	8.88	23.53	5.9	40.48	265.05
c24	TC	0	835.7	0	467.12	58.58	97.74	2.27	5.01	166.86

*YX, ‘Yunzheng Xiawei’ cultivar; SC, ‘Sun City’ cultivar; FW, fresh weight; SD, standard deviation; S, skewness; K, kurtosis; CV, coefficient of variation; TE, total epoxycarotenoids; TH, total hydroxycarotenoids; TX, total xanthophylls; THB, total hydrocarbons; TC, total carotenoids.*

### Sequencing Data and SNP Markers

In total, 33.48 and 43.85 Gb of raw data were obtained for the paternal and maternal lines, respectively. On average, 12.27 Gb of raw data were obtained for each offspring. The sequencing data were of sufficient quality, with an average Q30 percentage of 90.95%. The mapping rate of the male and female parents were 97.79 and 98.18%, respectively, indicating high-quality mapping results. After variant calling, a total of 16,038,514 initial SNPs were obtained from whole genome resequencing. These SNPs were first filtered, and those that were homozygous, had missing data or were of insufficient sequencing depth were removed. However, there were still too many SNPs remaining after screening (10,467,012). Thus, we used a sampling method to divide each chromosome into 10,000 regions according to the number and order of loci. Intermediate sites were selected within each region. Finally, 10,000 markers were obtained for each of the seven chromosomes. The pre-developed 440 SSR markers and 17,257 SNP markers obtained by SLAF-seq were then added, genotyped and imported into polymapR. During the mapping process, the markers were further filtered following the criteria within polymapR, and 78,438 markers were thus eliminated. Finally, a total of 9,259 high quality markers were obtained and mapped successfully.

**FIGURE 4:**
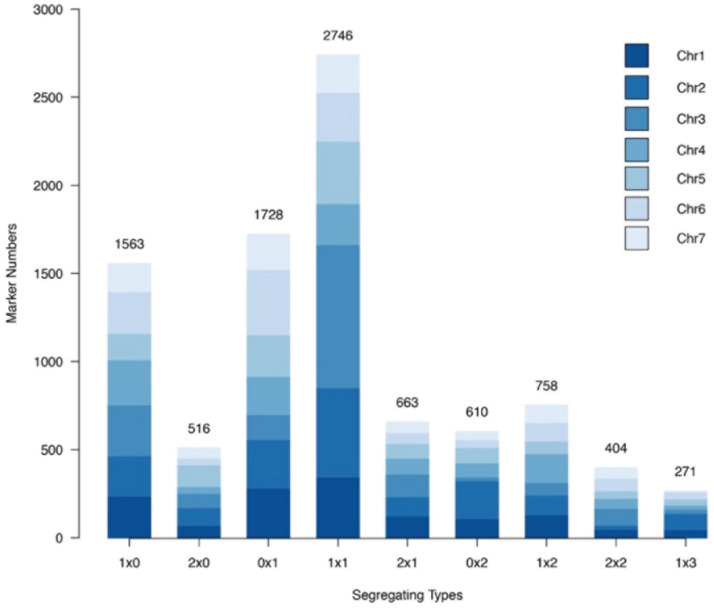
Number of each marker segregation type in the rose YS population among different chromosomes.

Nine different marker segregation types were identified, namely, 1 × 0, 0 × 1, 2 × 0, 0 × 2, 1 × 1, 1 × 2, 2 × 1, 1 × 3, and 2 × 2 (Figure 4). Type 1 × 1 segregation, spanning 2,746 markers, was the most common marker type, comprising 29.67% of all the markers mapped. The single-dose markers, type 1 × 0 and type 0 × 1 segregation patterns corresponding to 1,563 and 1,728 markers, respectively, together comprised 35.54% of all the markers. Type 2 × 2 and type 1 × 3 segregation patterns corresponded to the lowest numbers of markers, 404 and 271, respectively, comprising 4.36 and 2.93% of all markers.

### Linkage Map Construction

**FIGURE 5:**
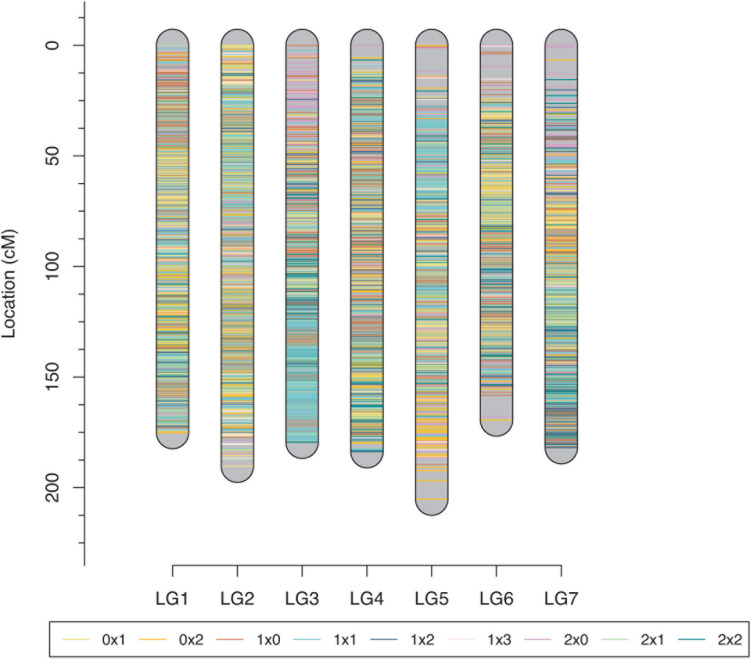
Final integrated map for the rose YS population. Different colors represent different segregating marker types.

The tetraploid rose genetic linkage map was constructed using polymapR with a total of 9,259 markers, including 175 SSRs and 9,048 SNPs. The final linkage map contained seven linkage groups (LGs), which is consistent with the number of haploid chromosomes observed in rose. The detailed map information is shown in Table 4 and Figure 5. The total length of the integrated map was 1285.11 cM, with an average distance of 0.14 cM between markers, indicating the high density of the map. The length of each LG ranged from 169.40 to 205.20 cM. LG2 and LG3 had the most markers, 1,669 and 1,653, respectively, with a minimum average distance of 0.11 cM. LG7 was the smallest linkage group, which contained a total of 949 markers separated by an average distance of 0.19 cM. Between two adjacent markers, the average percentage of gaps (≤5% total map length) was 99.9%, indicating the high coverage and even marker distribution of the linkage map.

**TABLE 4:** Summary of statistics for the linkage groups (LGs) of the genetic linkage map.

Linkage ID	Marker number	Distance (cM)	Average distance (cM)	Gap (≤5%)	Max gap (cM)	Marker linked to max gap
LG1	1405	175.10	0.12	100.00%	2.03	Chr1_50648541
LG2	1669	190.31	0.11	100.00%	3.20	Chr2_83190217
LG3	1653	179.46	0.11	100.00%	2.40	Chr3_41400110
LG4	1154	183.74	0.16	99.91%	5.60	Chr4_55969408
LG5	1186	205.20	0.17	99.83%	10.65	Chr5_58406380
LG6	1243	169.40	0.14	99.76%	9.68	Chr6_7025792
LG7	949	181.90	0.19	99.79%	6.48	Chr7_11747811
Total	9259	1285.11	0.14	99.90%	10.65	–

### QTL Analysis

**FIGURE 6:**
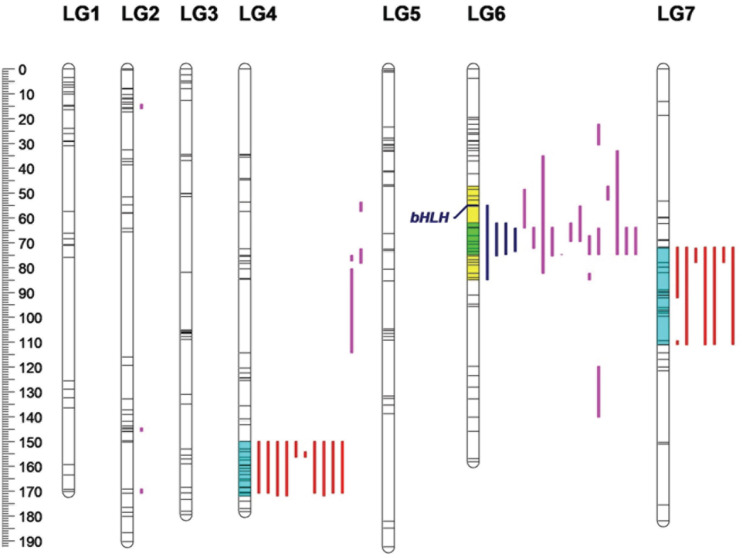
Locations of pigment content QTLs on the genetic linkage map of the rose YS population. Blue lines represent QTL regions associated with anthocyanin content; purple lines represent QTL regions associated with flavonol content, and red lines represent for QTL regions associated with carotenoid content.

A total of 67 anthocyanins, flavonols and carotenoids were analyzed by a whole-genome QTL analysis, and the detailed information is presented in Table 5. Significant QTLs were detected for 33 compounds, including four anthocyanins, 18 flavonoids and 11 carotenoids. Across the traits, 46 QTLs were detected, explaining 11.85–47.72% of phenotypic variation. The QTLs were primarily distributed on four linkage groups: LG2, LG4, LG6 and LG7 (Figure 6). Among them, the number of QTLs detected on LG6 (20) was the highest, with all of them contributing to variation in the flavonoid content; the numbers of QTLs on LG4 and LG7 were 15 and 8, respectively, and most of them were associated with variation in the carotenoid content. Three QTLs were identified on LG2.

**TABLE 5:** Summary of QTLs for pigment content traits detected in the YS population.

Traits		QTLs	LG	Peak Position (cM)	LOD thresholds	Markers	Confidence intervals (cM)	*R*^2^
**Anthocyanins**	antp2	qA2-6-1	6	68	8.49	Chr6_36524322	54–84	38.69%
	antp4	qA4-6-1	6	65	10.11	Chr6_52954641	62–75	34.59%
	antp7	qA7-6-1	6	65	13.26	Chr6_52954641	62–74	31.31%
	antp9	qA9-6-1	6	68	7.42	Chr6_36524322	64–73	45.97%
**Flavonols**	flap1	qF1-6-1	6	57	6.31	Chr6_56788295	48–64	18.73%
	flap3	qF3-6-1	6	71	6.32	Chr6_43233931	63–72	21.34%
	flap4	qF4-6-1	6	64	6.25	Chr6_60685803	34–70	15.64%
	flap8	qF8-6-1	6	74	7.16	Chr6_36324050	63–75	27.33%
	flap10	qF10-6-1	6	74	6.35	Chr6_36324050	74–75	38.90%
	flap11	qF11-6-1	6	67	6.87	Chr6_46802187	62–69	18.79%
		qF11-4-1	4	81	6.87	Chr4_18306051	80–82	30.93%
	flap12	qF12-6-1	6	55	6.20	Chr6_56716464	55–69	31.46%
	flap14	qF14-6-1	6	74	6.30	Chr6_36324050	67–74	23.79%
	flap15	qF15-6-1	6	78	6.35	Chr6_16827958	82–84	47.72%
	flap17	qF17-6-1	6	28	6.29	Chr6_68715376	23–30	16.66%
		qF17-4-1	4	87	6.29	Chr4_13499721	86–119	15.47%
	flap18	qF18-6-1	6	74	6.25	Chr6_36324050	65–74	23.56%
		qF18-4-1	4	61	6.25	Chr4_38841688	59–62	18.44%
	flap23	qF23-6-1	6	128	6.22	Chr6_33671102	119–140	43.49%
		qF23-2-1	2	169	6.22	Chr2_81248872	169–170	12.35%
	flap24	qF24-4-1	4	81	7.62	Chr4_18306051	78–83	17.42%
	flap25	qF25-2-1	2	16	6.35	Chr2_88416908	14–16	11.85%
	flap28	qF28-6-1	6	50	6.21	Chr6_64481122	46–53	13.84%
		qF28-2-1	2	145	6.21	Chr2_67825147	144–145	37.21%
	flap30	qF30-6-1	6	64	6.75	Chr6_60685803	33–74	17.54%
	flap31	qF31-6-1	6	74	6.32	Chr6_36324050	63–74	46.54%
	flap32	qF32-6-1	6	74	6.22	Chr6_36324050	63–74	46.62%
**Carotenoids**	carp2	qC2-4-1	4	156	7.65	Chr4_25116531	155–176	22.66%
	carp3	qC3-4-1	4	156	6.75	Chr4_25116531	155–176	24.15%
		qC3-7-1	7	73	6.75	Chr7_37223346	71–81	17.40%
	carp5	qC5-4-1	4	156	6.46	Chr4_25116531	155–176	22.13%
		qC5-7-1	7	109	6.46	Chr7_64982888	109–110	14.45%
	carp9	qC9-4-1	4	156	6.76	Chr4_25116531	155–177	20.39%
		qC9-7-1	7	73	6.76	Chr7_37223346	71–91	16.55%
	carp12	qC12-4-1	4	156	8.52	Chr4_25116531	155–177	20.06%
		qC12-7-1	7	73	8.52	Chr7_37223346	72–77	18.47%
	carp13	qC13-4-1	4	156	7.86	Chr4_25116531	155–161	23.81%
		qC13-7-1	7	74	7.86	Chr7_36167755	71–91	16.46%
	carp17	qC17-4-1	4	160	6.54	Chr4_52801971	159–161	16.34%
	carp20	qC20-4-1	4	156	6.74	Chr4_25116531	155–176	23.63%
		qC20-7-1	7	73	6.74	Chr7_37223346	71–92	17.05%
	carp21	qC21-4-1	4	156	7.23	Chr4_25116531	155–177	23.78%
		qC21-7-1	7	73	7.23	Chr7_37223346	71–77	17.51%
	carp22	qC22-4-1	4	156	6.76	Chr4_25116531	155–176	23.94%
		qC22-7-1	7	73	6.76	Chr7_37223346	71–92	17.33%
	carp24	qC24-4-1	4	161	6.55	Chr4_56663092	155–176	26.38%

Four QTL were detected for the anthocyanin content, all of which were distributed on LG6. The phenotypic variation rate ranged from 31.31 to 45.97%, which shows that all of them were major QTLs (i.e., contributing to more than 10% of the variation). The confidence intervals of the four loci highly overlapped. Among them, qA9-6-1 had the highest contribution rate, accounting for 45.97% of variation. The molecular marker closely linked to qA9-6-1 was Chr6_36524322.

Additionally, 23 QTLs were detected for flavonol contents in the three linkage groups. Among them, 16 QTLs were found on LG6. On LG2 and LG4, there were three and four QTLs, respectively. The phenotypic variation rate ranged from 11.85 to 47.72%. Among the 23 QTLs, 12 and eight QTLs explained more than 20 and 30% of phenotypic variation, respectively. Among the detected loci, qF15-6-1 had the rate of highest contribution, and its closely linked molecular marker was Chr6_16827958.

A total of 19 QTLs were identified to be associated with the content of carotenoid, which were distributed in two linkage groups. The number of QTLs on LG4 and LG 7 were 11 and eight, respectively, and they explained from 14.45 to 26.38% of the phenotypic variation. The 19 loci had highly overlapping confidence intervals. Most had the same QTL peaks and linked markers, indicating that many carotenoids were controlled by the same QTLs. Among the loci detected, qC24-4-1 had the highest contribution rate to phenotypic variation, and its closely linked molecular marker was Chr4_56663092.

## Discussion

### Tools for Genetic Studies in Polyploids

Polyploid species follow a very complicated pattern of meiosis that differs from the pattern in diploid organisms. Therefore, it is very difficult to conduct genetic studies of polyploids. As more research in polyploid species is conducted, the necessary tools are also being developed, thus, rendering the genetic analysis of polyploid populations progressively more reliable.

The number of tools for polyploid linkage mapping is continuously increasing, from the well-known TetraploidMap (Hackett et al., 2007), to recently developed R packages, such as PERGOLA (Grandke et al., 2017), polymapR (Bourke et al., 2018b), and MApoly (Mollinari and Garcia, 2019), among others. More tools have been developed to focus on a greater number of population types and multiple ploidy levels. For example, PolymapR, which was used in this study, can perform linkage analysis for polysomic triploids, tetraploids and hexaploids, as well as segmental allotetraploid populations (Bourke et al., 2018c). The polyqtlR R package is a reliable tool for QTL analysis using identity-by-descent (IBD) probabilities in F_1_ populations of outcrossing in heterozygous polyploid species. With the continual analysis of polyploid genetic model systems (Jiang et al., 2020; Sun et al., 2020), the software tools for polyploid genetic research will also provide a better understanding of polyploid inheritance.

### High Density Linkage Map Construction

Linkage map construction for roses has primarily been focused at the diploid and tetraploid levels. The first rose genetic linkage map was constructed by Debener and Mattiesch (1999) using a diploid *Rosa multiflora* population. Subsequently, more diploid rose maps using different markers have been published (Debener et al., 2001; Crespel et al., 2002; Dugo et al., 2005; Yan et al., 2005, 2018; Linde et al., 2006; Hibrand Saint-Oyant et al., 2008; Remay et al., 2009; Spiller et al., 2011; Li et al., 2019; Lopez Arias et al., 2020). Studies of tetraploid populations are relatively less common than those of diploid rose populations. Therefore, our study aimed to increase the quality of tetraploid rose genetic map by improving the map length, density and coverage, thus, providing a solid foundation for the fine mapping of quantitative traits, map-based gene cloning and functional gene mining of tetraploid roses in the future.

Most of the published linkage maps of roses are based on traditional molecular markers, such as AFLPs, restriction fragment length polymorphisms (RFLPs) and SSRs. However, markers developed by sequencing technology have gradually replaced traditional markers. With the advantages of higher coverage and substantially higher marker number, the length and density of genetic maps can be significantly improved. Yan et al. (2018) generated the first diploid rose genetic map based on genotyping by sequencing technology data. They mapped 20 SSRs and 3,507 SNPs onto a total map length of 892 cM, with an average gap of 0.25 cM. However, they used *Fragaria vesca* as the reference genome, and that may have reduced the accuracy of SNP calling. Li et al. (2019) constructed a diploid rose genetic map using RAD-seq technology. The map was improved in terms of its length, which was 1027.4 cM, but the marker number and map density decreased, with an average distance of 0.96 cM between adjacent markers. Compared with reduced-representation genome sequencing (RRGS), whole-genome resequencing has a wider genome coverage and can thus, obtain more comprehensive genomic information. WGS technology has been widely used in crops, such as peanut (Agarwal et al., 2018), watermelon (Guo et al., 2019), cucumber (Wang et al., 2019), and others. However, this approach has not been widely used in rose research.

In previous research, the YS population was used to construct a genetic map using SLAF-seq technology (Yu et al., 2021) (Table 6). A total of 6,842 markers were contained in a 1,158.90 cM map, with an average distance of 0.18 cM between markers. However, the maximum gap was 29.16 cM, indicating that the distribution of markers was uneven. Based on the previous map, we made further improvements using WGS technology. In this new ultra-high-density genetic map, a total of 9,259 molecular markers were well-distributed over seven LGs. The map has achieved substantial improvements in both length and density, with a total length of 1,285.11 cM and an average marker spacing of 0.14 cM. This improved map can serve as a strong tool for rose genetic research, as well as the marker-assisted breeding of tetraploid roses.

**TABLE 6:** Comparison between maps constructed by the specific locus amplified fragment sequencing (SLAF-seq) and whole-genome sequencing (WGS) methods across linkage groups (LG).

Method	Number of markers								Total distance (cM)	Average distance (cM)	Max gap (cM)
	LG1	LG2	LG3	LG4	LG5	LG6	LG7	Total			
SLAF-seq	775	1248	827	764	858	898	1472	6842	1158.90	0.18	29.16
WGS	1405	1669	1653	1154	1186	1243	949	9259	1285.11	0.14	10.65

### Mapping Flower Color Related Traits

The improvement of flower color is of substantial significance to the ornamental value and economic value of roses. A substantial amount of research has been conducted on pigment contents, compositions and variations in roses. Schmitzer et al. (2009) studied the pigment composition of modern rose petals and found that the main anthocyanins were cyanidin 3,5-dioxyglucoside and geranium 3,5-dioxyglucoside, and the secondary anthocyanins were cyanidin 3-*O*-glucoside and geranium 3-*O*-glucoside. These four anthocyanins were also detected and mapped in F1 population in this study, and all of them were located on LG4. The flavonols detected in this study, kaempferol 3-*O*-rutinoside, kaempferol 3-*O*-glucoside and kaempferol, are widely found in the petals of *R. chinensis*, *Rosa gallica*, *Rosa Rugosa*, and *Rosa damascena* (Kumar et al., 2009; Sarangowa et al., 2014). This proved that the results of this study are precise and accurate. Compared with anthocyanins and flavonols, studies on carotenoids in Rosa petals have been studied less frequently.

In this study, we detected a total of 46 QTLs related to pigment content traits based on the newly developed linkage map. The phenotypic variation associated with these QTLs was greater than 10% for all the traits, indicating that all of them were major QTLs. The LOD thresholds obtained from the QTL analysis were high, ranging from 6.20 to 13.26. This may result in the omission of some QTLs with smaller effects but can effectively reduce the detection of false-positive QTLs, which is necessary for further fine mapping and map-based cloning.

Many studies have mapped flower color traits of diploid and tetraploid rose populations, but most have focused on chromatism. Few studies have analyzed anthocyanins and mapped major QTLs across several linkage groups. Henz et al. (2015) analyzed total anthocyanins in diploid rose progeny under different environments, detecting two major QTLs on ICM2 and ICM6 in all the environments. A marker derived from a *bHLH* (basic helix-loop-helix) gene was mapped to the center of ICM6, which corresponds to our current results (Figure 6). Gitonga et al. (2016) also detected QTLs on ICM6 in the tetraploid K5 population. In our study, all of the variation in content of anthocyanin compounds was mapped to LG6, with peak positions around 65 cM. This is consistent with previous studies and showed that *bHLH* is significantly associated with anthocyanin biosynthesis. Moreover, most flavonols also mapped to LG6, and the confidence intervals overlapped with the range of anthocyanins. It is assumed that these traits are controlled by the same genes in the flavonoid metabolism pathway and have similar genetic bases.

Few studies have mapped loci associated with carotenoids in roses. Schulz et al. (2016) presented a genome-wide association study (GWAS) for tetraploid roses and mapped anthocyanin and carotenoid contents. However, because of the lack of a published rose genome, they were limited to mapping the traits to the *Fragaria* and *Prunus* genomes. In early studies, Yu et al. (2021) used a previous map developed by SLAF-seq technology to locate QTLs associated with carotenoid contents and found that there were QTL clusters on both LG4 and LG7 (Table 6), which is also consistent with the current results. However, in previous studies, QTLs were analyzed by interval mapping. Thus, the results were not as accurate or comprehensive as the results of composite interval mapping in this study.

QTLs are easier to map to the same region if the traits are highly correlated with each other (Guan et al., 2015). The QTLs mapped in this study were clustered, and the QTLs for different carotenoids were located in roughly the same regions of LG4 and LG7. Santos and Simon (2002) also found that multiple QTLs that controlled the content of β-carotene and ζ-carotene were clustered together, which may be owing to the fact that related phenotypic traits are regulated by one or a few pleiotropic gene. Thus, they can effectively improve the utilization of genes and reduce the loss of favorable allele combinations through recombination.

In this study, the tetraploid rose genetic map with the highest density was constructed by combining genomic and metabolomic techniques, and QTL mapping of metabolites related to flower color was conducted. Integrating genomics, metabolomics and other omics data to analyze the synthesis pathways of secondary metabolites can assist modern bioengineering technology to improve the content of related pigments in ornamental plants and promote the comprehensive utilization of edible and medicinal plant materials. The results of this study provide the basis for the fine mapping of flower color genes in roses and thus, suggest new objectives for the production of natural metabolites and the development of rose products.

## Data Availability Statement

The raw data supporting the conclusions of this article will be made available by the authors, without undue reservation.

## Author Contributions

BC performed the efinal data analysis and wrote the manuscript. HW collected and analyzed the phenotypic data. YH, CY, and LL helped to improve the method and edited the manuscript. CY, HP, and QZ participated in supervision and helped to revise the manuscript. All authors contributed to the article and approved the submitted version.

## Conflict of Interest

The authors declare that the research was conducted in the absence of any commercial or financial relationships that could be construed as a potential conflict of interest.
