# Supplementary figures and images for: Identification and QTL Analysis of Flavonoids and Carotenoids in Tetraploid Roses Based on an Ultra-High-Density Genetic Map
Source: Front Plant Sci. 2021 Jun 11;12:682305. doi: 10.3389/fpls.2021.682305 (PMC8226220; doi:10.3389/fpls.2021.682305)

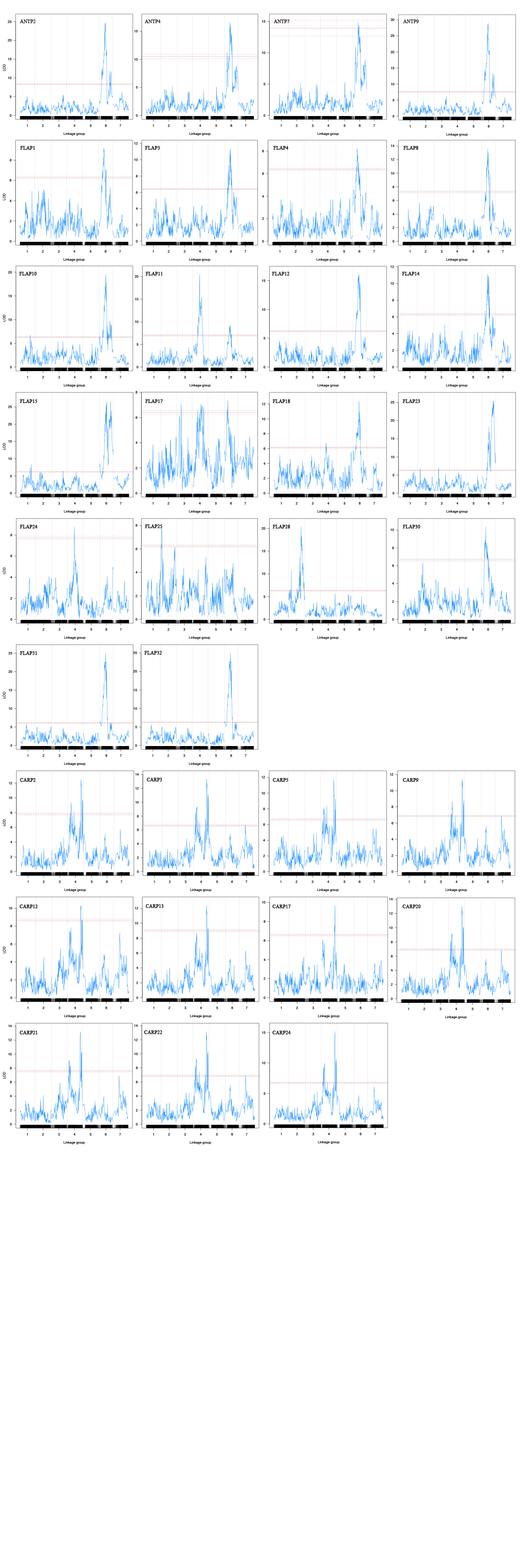

Supplement: Supplementary file 1 [file Image_1.png]
